# Chikungunya virus requires cellular chloride channels for efficient genome replication

**DOI:** 10.1371/journal.pntd.0007703

**Published:** 2019-09-04

**Authors:** Marietta Müller, Natalie Slivinski, Eleanor J. A. A. Todd, Henna Khalid, Raymond Li, Magdalena Karwatka, Andres Merits, Jamel Mankouri, Andrew Tuplin

**Affiliations:** 1 School of Molecular and Cellular Biology, Faculty of Biological Sciences and Astbury Centre for Structural and Molecular Biology, University of Leeds, Leeds, United Kingdom; 2 Institute of Technology, University of Tartu, Tartu, Estonia; WRAIR, UNITED STATES

## Abstract

Chikungunya virus (CHIKV) is a re-emerging, pathogenic alphavirus that is transmitted to humans by *Aedes spp*. mosquitoes—causing fever and debilitating joint pain, with frequent long-term health implications and high morbidity. The CHIKV lifecycle is poorly understood and specific antiviral therapeutics or vaccines are lacking. In this study, we investigated the role of host-cell chloride (Cl^-^) channels on CHIKV replication.We demonstrate that specific pharmacological Cl^-^ channel inhibitors significantly inhibit CHIKV replication in a dose-dependent manner, suggesting that Cl^-^channels are pro-viral factors in human cells. Further analysis of the effect of the inhibitors on CHIKV attachment, entry, viral protein expression and replicon replication demonstrated that Cl^-^ channels are specifically required for efficient CHIKV genome replication. This was conserved in mosquito cells, where CHIKV replication and genome copy number was significantly reduced following Cl^-^ channel inhibition. siRNA silencing identified chloride intracellular channels 1 and 4 (CLIC1 and CLIC4, respectively) as required for efficient CHIKV replication and protein affinity chromatography showed low levels of CLIC1 in complex with CHIKV nsP3, an essential component of the viral replication machinery. In summary, for the first time we demonstrate that efficient replication of the CHIKV genome depends on cellular Cl^-^ channels, in both human and mosquito cells and identifies CLIC1 and CLIC4 as agonists of CHIKV replication in human cells. We observe a modest interaction, either direct or indirect, between CLIC1 and nsP3 and hypothesize that CLIC1 may play a role in the formation/maintenance of CHIKV replication complexes. These findings advance our molecular understanding of CHIKV replication and identify potential druggable targets for the treatment and prevention of CHIKV mediated disease.

## Author summary

Chikungunya virus (CHIKV) is a mosquito-borne virus that infects humans and causes chikungunya fever—characterized by fever, rash and chronic arthralgia. Treatment of chikungunya fever is limited to the alleviation of the symptoms and no vaccine is available to prevent infection. Consequently, new anti-CHIKV therapies or targets are urgently required. Since its initial isolation in Tanzania in 1952, CHIKV has spread widely across tropical/sub-tropical and more temperate regions. It has been responsible for epidemics in >70 different regions of the world, resulting in high morbidity and financial burden. Despite this, the CHIKV lifecycle is poorly understood. Here, we demonstrate for the first time that CHIKV requires host-cell ion channels that mediate chloride ion (Cl^-^) flux through the membranes of infected cells, to complete its lifecycle. Specifically, using pharmacological compounds, we show that Cl^-^ channels are required for efficient replication of the virus genome, identify two specific channels (CLIC1 and CLIC4) required for replication and demonstrate that CLIC1 may interact with viral non-structural protein 3 (nsP3)—an essential component of the CHIKV replicase complex. These findings advance our understanding of CHIKV replication and identify potential druggable targets for the treatment and prevention of CHIKV mediated disease.

## Introduction

Chikungunya virus (CHIKV) is a mosquito-borne virus of the *Alphavirus* genus in the *Togaviridae* family. It was first isolated during an outbreak in Tanzania in 1952 [1], since which its geographic range has expanded globally to include almost 40 countries [2]. Following transmission, CHIKV replicates in the fibroblasts of the dermis and disseminates through the blood-stream to several tissues including muscle, joints and the liver [3]. CHIKV causes chikungunya fever which is characterized by high fever, maculopapular rash, myalgia and debilitating arthralgia [4]. In some cases more severe symptoms occur—including encephalitis, encephalopathy, myocarditis, hepatitis and Guillain Barré syndrome [5, 6], however chikungunya fever-associated death is rare [7]. Due to the chronic debilitating symptoms and sequela that persist for up to 3 years [8], CHIKV has a major impact on morbidity and loss of economic productivity within at-risk populations [9]. Treatment of CHIKV-associated disease is limited to the relief of symptoms with no licensed vaccines or direct acting antivirals currently available.

CHIKV is a small, enveloped virus, with a single-stranded positive-sense RNA genome of ~12 kilobases. The genome possesses a type-0 5’ 7-methyl-GpppA cap, a 3’ poly(A) tail and two open reading frames (ORFs). The first ORF (ORF-1) encodes the non-structural polyprotein P1234 that is processed to yield four mature non-structural proteins (nsP1-4). The second ORF (ORF-2) encodes the structural polyprotein that is processed into the capsid protein, E3, E2, 6K, and E1. Virus attachment onto mammalian cells is mediated by the CHIKV E2 protein and the cell adhesion molecule Mxra8 [10]. Internalization is achieved via clathrin-mediated endocytosis, although clathrin-independent pathways have also been identified [10, 11]. Following CHIKV trafficking to early endosomes, the E1 glycoprotein facilitates endosomal fusion and capsid release into the cytoplasm [11, 12]. Replication of the viral genome is not well studied but through analogy to other alphaviruses proteolytic cleavage of P1234 in *cis* by the protease function of nsP2 releases the RNA-dependent RNA polymerase nsP4, initiating the synthesis of minus-strand RNA. Subsequent proteolytic cleavage of the remaining P123 polyprotein initiates synthesis of genomic and sub-genomic RNAs from the minus-strand template [13]. Viral RNA replication occurs in membrane-bound replication complexes, termed spherules, located at the plasma membrane [13, 14], facilitating an optimal environment for replication and protection of dsRNA intermediates from host cell detection. The structural polyprotein is translated from sub-genomic RNA and is co- and post- translationally cleaved by viral and host proteases. Virus assembly and budding takes place at the plasma membrane.

The regulation of ionic homeostasis, mediated through cellular ion channels, has emerged as a requirement for a number of virus infections [15]. It has previously been shown that two pore domain (K_2P_) potassium channels play a role during the trafficking of Bunyamwera virus (family *Peribunyaviridae)* in endosomes [16, 17] and that Hazara virus (family *Nairoviridae)* similarly requires endosomal potassium to permit release from endosomes [18]. Ebola virus (family *Filoviridae*) is known to require endosomal calcium channels for entry into its host cells [19], while entry of Influenza virus (family *Orthomyxoviridae*) depends on binding of the hemagglutinin to the voltage-dependent calcium channel Ca_v_1.2 [20]. Hepatitis C virus (family *Flaviviridae*) replication is inhibited by Cl^-^ channel blockers [21] whilst the Cl^-^ channel CLC6 was identified as a pro-viral factor in a genome-wide loss of function screen during CHIKV infection [22].

In this study, we used a panel of ion channel modulating compounds to demonstrate that CHIKV requires Cl^-^ channel activity during its lifecycle, in both mammalian and mosquito cells, for efficient replication of the viral genome. Through RNAi silencing, two Cl^-^ intracellular channels, CLIC1 and CLIC4, were identified as pro-viral factors for CHIKV replication, with CLIC1 potentially interacting with CHIKV nsP3—further implicating this channel as a significant host cell factor during CHIKV infection. These findings expand our understanding of CHIKV pathogenesis and reveal Cl^-^ channels as a potential host cell target for the development of much needed CHIKV antivirals.

## Materials and methods

### Cells and viruses

Huh7 cells (hepatocytes derived from human hepatocellular carcinoma) and BHK-21 cells (fibroblasts derived from Syrian golden hamster kidney) were a gift from M. Harris (University of Leeds, UK). C6/36 cells (*Aedes albopictus* larva) were a gift from S. Jacobs (The Pirbright Institute, UK). All cell lines tested negative for mycoplasma. Mammalian cells were maintained in Dulbecco’s modified Eagle’s medium (DMEM, Sigma) supplemented with 10% fetal bovine serum, 100 units/ml penicillin, 100 μg/ml streptomycin and non-essential amino acids (Lonza) in a humidified incubator at 37°C and 5% CO_2_. Invertebrate cells were maintained at 28°C in Leibovitz’s media (Gibco) supplemented with Tryptose Phosphate Broth (Thermo Fisher Scientific) and 100 units/ml penicillin, 100 μg/ml streptomycin.

The CHIKV ICRES and CHIKV-Fluc replicon (SGR), in which the ORF-2 region encoding the structural proteins has been replaced with sequence encoding firely luciferase, cDNA clones were previously described [23]. Both are based on the isolate LR2006 OPY1, representing the East Central South African genotype. CHIKV TST-nsP3 was generated by synthesizing a 129 bp DNA fragment containing the twin-strep-tag (TST) encoding sequence flanked by two SpeI restriction sites (ThermoFisher Scientific). This fragment was cloned into the CHIKV-Fluc SGR using a unique SpeI site in region corresponding to the hypervariable domain of nsP3 (position 5222). The TST-nsP3 fragment was then excised and cloned into the CHIKV ICRES plasmids using the unique KfII and the AgeI restriction sites. All CHIKV plasmids were linearized using NotI and *in vitro* transcribed using the mMESSAGE mMACHINE SP6 Kit (Ambion). 1 μg of RNA was electroporated into 1.2 × 10^6^ BHK-21 cells using a single square wave pulse (260 V, 25 ms). Cells were seeded into a 75cm^2^ flask and incubated for 48 hrs at 37°C. Supernatant was harvested, clarified by centrifugation for 5 min at room temperature (RT), aliquoted and stored at -80°C. The CHIKV titer was determined by standard plaque assay on BHK-21 monolayers and expressed as plaque forming units/ml (PFU/ml).

### Determination of maximum-non-toxic dose (MNTD) of synthetic compounds

Huh7 cells were seeded into 96 well plates at 10 000 cells/well (20 000 cells/well for C6/36 cells) one day prior to treatment with increasing doses of Ribavirin (Fluorochem); 4,4'-Diisothiocyanato-2.2'-stilbenedisulfonic acid (DIDS, Sigma); 9-Anthracenecarboxylic acid (9-ACA, Sigma); indanyloxyacetic acid-94 (IAA-94, Cayman Chemical), 5-Nitro-2-(3-phenylpropylamino)benzoic acid (NPPB, Santa Cruz Biotechnology) and dimethyl sulfoxide (DMSO, Fisher Chemical). Compound stocks were dissolved in DMSO and stored at -20°C in aliquots until dilution into complete DMEM/Leibovitz’s for addition onto cells. After 6 hrs and 24 hrs incubation respectively, media/compound was removed and cells incubated in Opti-MEM (Gibco) plus 1 mg/ml 3-(4,5-Dimethylthiazol-2-yl)-2,5-Diphenyltetrazolium Bromide (MTT) for 30 min at 37°C. After removal of Opti-MEM/MTT, cells were lysed in DMSO and absorbance determined at 570 nm on an Infinite F50 microplate reader (Tecan). Absorbance of samples was normalized to untreated control cells. Three independent experiments were conducted, each repeat consisting of three wells/dose. A two-way ANOVA was employed and a Sidak’s or Dunnett’s multiple comparisons test was performed, comparing each compound dose to the average of untreated samples. The MNTD was defined as the highest compound concentration that does not show a significant reduction in normalized absorbance to the untreated samples.

### Treatment of mammalian and invertebrate cells with compounds and infection with CHIKV

Huh7 cells were treated with MNTDs of Ribavirin, DIDS, 9-ACA, IAA-94, NNPB and DMSO (control) as follows. Huh7 cells were seeded into 12 well plates at 100 000 cells/well. The next day, CHIKV at MOI 2 (2xPFU as determined on BHK-21 monolayers/Huh7 cell number) and the respective inhibitor were diluted in 150 μl complete DMEM and adsorbed to the cells for 1 hr at 37°C. CHIKV/compound was removed, cells washed with PBS and incubated for 12 hrs in DMEM/compound. Supernatant was then harvested the titer of released CHIKV determined by plaque assay. To determine the virucidal activity of the compounds, the inhibitors and CHIKV were diluted into 150 μl with complete DMEM and incubated at 37°C for 1 hr prior to adsorption to the cells. Treatment with DEPC was conducted by diluting CHIKV and DEPC (2 mM final concentration) in PBS and incubating at RT in the dark. CHIKV and compounds were adsorbed to cells for 1 hr at 37°C and were then removed and cells incubated for 12 hpi in complete DMEM. The supernatants were harvested and CHIKV titer determined by standard plaque assay. The impact of the compounds on CHIKV attachment to the cell surface was investigated by performing CHIKV adsorption in the presence of the inhibitors at 4°C for 1 hr to prevent uptake. Cells were then rigorously washed with cold PBS and returned to 37°C in complete DMEM. At 24 hpi supernatants were harvested and CHIKV titer determined by standard plaque assay at 24 hpi. The first two hours of the CHIKV lifecycle were investigated by treating the cells with the inhibitors in 1 ml DMEM 1 hr prior to absorbing the virus in the presence of the inhibitors. After removal of the virus, cells were incubated one further hour in 1 ml complete DMEM plus inhibitors before these were removed by washing in PBS. Cells were then kept in complete DMEM until the titer of the released virus was determined at 12 hpi.

C6/36 cells were seeded into 12 well plates at 200 000 cells/well. The next day, cells were infected with CHIKV at MOI 2 (2xPFU as determined on BHK-21 monolayers/C6/36 cell number) and treated with the MNTD of compounds as described for Huh7 cells. The supernatants were harvested and CHIKV titer determined at 24 hpi. All experiments were performed in three independent repeats, each consisting of 2 wells/condition. A one-way ANOVA was employed and a Dunnett’s multiple comparisons test was performed, comparing treated samples to untreated sample.

### Assessment of viral protein expression by western blot

Huh7 cells were seeded into 6 well plates at 300 000 cells/well. The next day, cells were infected with CHIKV (MOI 0.1, 2, 10) in the presence of the MNTD of the inhibitors as described above. After removal of the virus, cells were incubated in complete DMEM/inhibitor until cells were washed in PBS, detached by trypsinization, and lysed in lysis buffer (25 mM Tris•HCl pH 7.4, 150 mM NaCl, 1% NP-40, 1 mM EDTA, 5% glycerol) at 24 hpi. Cell lysate was cleared by centrifugation at >16 0000 x g for 10 mins at 4°C and total protein concentration determined by Pierce BCA Protein Assay (Thermo Scientific). Equal amounts of protein were resolved by SDS-PAGE and proteins transferred to PVFD membranes using semi-dry transfer. Western blots were probed with antibodies against nsP1 (1:1000, rabbit polyclonal, in-house)[24], nsP3 (1:1000, rabbit polyclonal, in-house)[24, 25], capsid (1:1000, rabbit polyclonal, in-house), CLIC1 (1:1000, mouse monoclonal, Abcam, ab77214), CLIC4 (1:200, mouse monoclonal, Santa Cruz Biotechnology, sc-135739) and the housekeeping protein actin (clone AC-15, mouse monoclonal, Sigma) overnight at 4°C diluted in Odyssey Blocking Buffer (Li-Cor). After washing in PBS, the western blot was incubated for 1 hr at RT with the respective secondary antibodies (IRDye 800CW Donkey anti-Mouse; IRDye 680LT Donkey anti-Rabbit; Li-Cor). The membrane was dried an imaged using the Odyssey Fc Imaging System (Li-Cor).

### Quantification of Clathrin-mediated endocytosis

Huh7 cells were seeded into 12 well plates at 100 000 cells/well. The next day, cells were washed and incubated in live cell imaging solution (140 mM NaCl, 2.5 mM KCl, 1.8 mM CaCl_2_, 1 mM MgCl_2_, 20 mM HEPES pH 7.4, 20 mM Glucose, 1% BSA) supplemented with the MNTD of inhibitory compounds for 50 mins at 37°C. Cells were incubated on ice for 10 mins and Alexa Fluor 488 EGF complex (Invitrogen E13345) was added to a final concentration of 0.8 μg/ml. After 45 mins, cells were fixed in 4% Formaldehyde/PBS and imaged using the IncuCyte ZOOM system (Essen Bioscience). The default software parameters for a 12 well plate (Corning) with a 10× objective was used for imaging 4 fields of view/well. A processing definition was established to automate identification of Alexa Fluor 488 positive objects. The green object count/well was extrapolated by the IncuCyte ZOOM software. Two wells/conditions were analyzed in three biological repeats. A one-way ANOVA was employed and Dunnett’s multiple comparisons test was performed comparing each sample to the untreated sample. A modest, but significant decrease in Alexa Fluor 488 EGF uptake upon treatment with the carrier control (DMSO) was controlled for by additionally comparing the samples to DMSO-treated samples in the Dunnett’s multiple comparison test.

### SGR and luciferase assays

The CHIKV-Fluc SGR contains a Firefly Luciferase (Fluc) reporter gene which is expressed from the sub-genomic promotor [23]; therefore, detection of Fluc activity is indicative of CHIKV genome replication. The plasmid was linearized using NotI and *in vitro* transcribed using the mMESSAGE mMACHINE SP6 Kit (Ambion). 400 ng CHIKV-Fluc SGR RNA was co-transfected with 100 ng Renilla Luciferase (Rluc) encoding RNA using 1 μl Lipofectamine 2000 (Thermo Fisher) into Huh7 cells seeded the previous day into 24 well plates at 50 000 cells/well. During the time of transfection, cells were incubated with Opti-MEM/inhibitor. Cells were lysed in passive lysis buffer (Promega) at 6 hrs post-transfection. The dual luciferase assay was performed according to the manufacturer’s protocol (Promega). The Fluc signal was adjusted to the Rluc signal as follows: (average Rluc signal (untreated control)/ Rluc signal (sample X)) x Fluc signal (sample X). Three independent repeats were performed, with each repeat consisting of 2 wells/conditions, which were analyzed as 2 technical repeats each. A one-way ANOVA was employed and Dunnett’s multiple comparisons test was performed comparing each sample to the untreated sample.

### Strand-specific Quantification of CHIKV RNA

Huh7 cells were infected with CHIKV and treated with inhibitory compounds as described above. At 6 hpi, total RNA was extracted from cells using TRI Reagent Solution (Applied Biosystems) according to the manufacturer’s instructions. Strand-specific qPCR (ssqPCR) was performed according to the protocol described by Plaskon and colleagues [26]. Briefly, 500 ng of RNA were reverse-transcribed with gene specific primers (S1 Table) using the SCRIPT cDNA Synthesis Kit (Jena Bioscience) according to the manufacturer’s protocol. 100ng of strand-specific cDNA was used as template for the quantitative PCR performed with the qPCRBIO SyGreen Blue Mix Lo-ROX (PCR Biosystems) with gene specific primers (S1 Table) amplifying a 94 bp region of the CHIKV nsP1 encoding sequence using the following PCR program: 95°C for 2 mins, 40 x (95°C for 5 sec, 60°C for 30 sec), dissociation curve 60°C-95°C as pre-defined by the Mx3005P thermal cycler (Agilent technologies). *In vitro* transcribed CHIKV ICRES RNA was reverse transcribed and a cDNA dilution series employed as a standard to quantify copy numbers in the respective samples. All experiments were performed in four independent repeats, each consisting of 2 wells/condition. A one-way ANOVA was employed and Dunnett’s multiple comparisons test was performed comparing each sample to the untreated control sample.

### Quantification of CHIKV genome copy numbers

C6/36 cells were infected with CHIKV (MOI 2) and treated with inhibitory compounds as described above. At 24 hpi, total RNA was extracted from cells using TRI Reagent Solution (Applied Biosystems) according to the manufacturer’s instructions. RNA integrity was confirmed by denaturing ageraose gel electrophoresis before reverse transcription of 1 μg of RNA using the High-Capacity RNA-to-cDNA Kit (Applied Biosystems) according to the manufacturer’s protocol. Quantitative PCR was performed using the qPCRBIO SyGreen Blue Mix Lo-ROX (PCR Biosystems) with primers amplifying a 78 bp region of the CHIKV nsP1 encoding sequence (fwd primer: 5’CCGACTCAACCATCCTGGAT’3, rev primer: 5’GGCAGACGCAGTGGTACTTCCT’3), 100ng of cDNA template and a PCR program as described above. *In vitro* transcribed CHIKV ICRES RNA was reverse transcribed and a cDNA dilution series employed as a standard to quantify copy numbers in the respective samples. All experiments were performed in three independent repeats, each consisting of 2 wells/condition. A one-way ANOVA was employed and Dunnett’s multiple comparisons test was performed, comparing each sample to the untreated control sample.

### siRNA mediated knock down of CLIC1 and CLIC4

Huh7 cells were seeded into 12 well plates at 100 000 cells/well. The next day, cells were transfected with 75 pmol/well CLIC1, CLIC4 and control-B (= scrambled) siRNA (Santa Cruz Biotechnology), respectively, with each siRNA representing a pool of three 19–25 nt siRNAs. Transfection was conducted according to the manufacturer’s protocol (Santa Cruz Biotechnology sc-29528) with a siRNA to transfection reagent ratio of 1:1. At 24 hrs post transfection, the transfection was repeated to achieve a clearly discernable reduction in protein expression level. At 48 hrs post initial transfection, cells were infected with CHIKV (MOI 10) as described above and a sample harvested for analysis on western blot. Supernatant was harvested at 24 hpi and standard plaque assays performed to determine viral titer. Cells were lysed and analyzed on western blots as described. For double knock down of CLIC1 and CLIC4, a total of 150 pmol siRNA/well was transfected into the cells. Three independent repeats were performed and CHIKV titers of CLIC1/CLIC4 knock down samples were expressed as percentage of scrambled siRNA control sample per repeat. A one-way ANOVA was employed and Dunnett’s multiple comparisons test was performed, comparing each sample to the scrambled siRNA control sample.

### Strep-Tactin affinity chromatography

Huh7 cells were infected with CHIKV ICRES TST-nsP3 (MOI 10) as described above. At 24 hpi, cells were lysed in lysis buffer (2x, pH 7.2, 20mM PIPES, 240mM KCl, 60mM NaCl, 10mM MgCl2, 2% Triton x-10, 20% Glycerol) and total protein concentration of clarified cell lysate determined by BCA assay (Pierce). Strep-Tactin Sepharose 50% suspension (iba, 2-1201-002) was washed with wash buffer (100 mM Tris-Cl, 150 mM NaCl, 1 mM EDTA; pH 8) before equilibrating with lysis buffer. 1mg/ml total protein was incubated with the sepharose o/n at 4°C. Sepharose was washed once with lysis buffer followed by wash buffer (100 mM Tris/HCl, pH 8.0; X mM NaCl; 1 mM EDTA) with increasing and then decreasing stringency (150 mM, 275 mM, 500 mM, 150 mM NaCl). Sepharose was boiled in standard SDS sample buffer and bound proteins analyzed on western blots as described above.

## Results

### Inhibition of Cl^-^ channels decreases CHIKV replication

**Fig 1:**
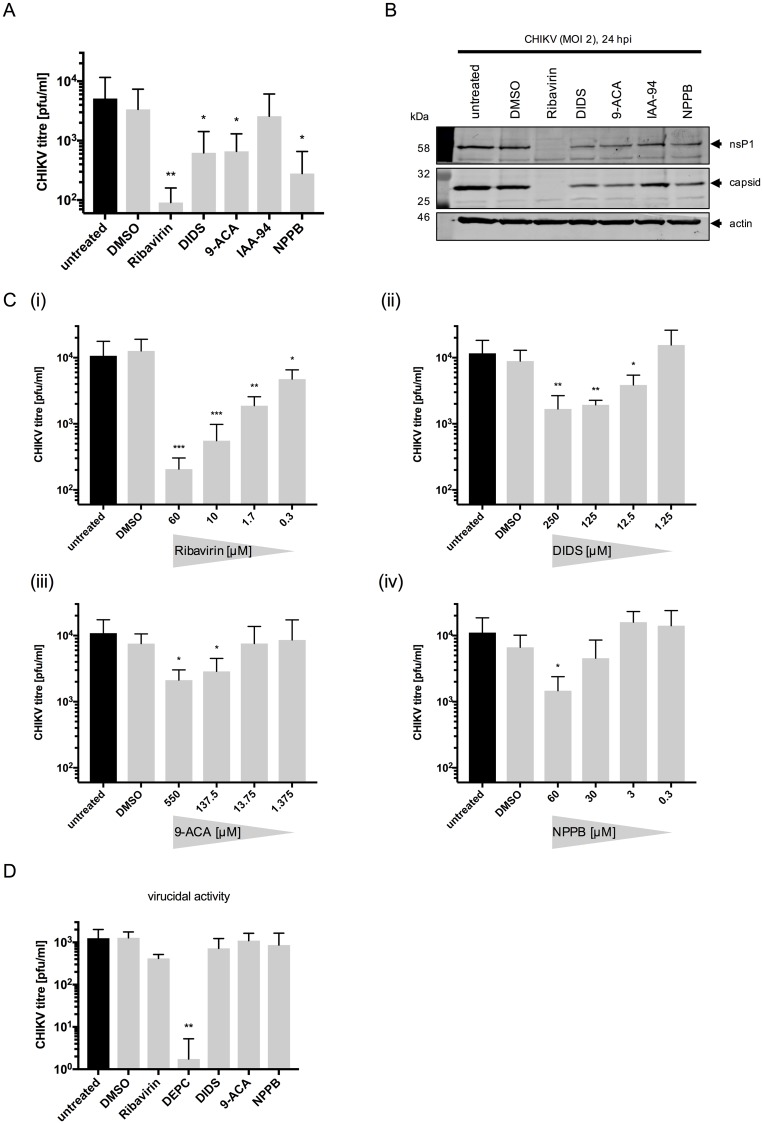
Inhibition of Cl^-^ channels decreases CHIKV replication. **A)** Huh7 cells were infected with CHIKV (MOI 2) in the presence of the MNTD of Cl^-^ channel inhibitors; Ribavirin served as a positive control and DMSO as a carrier control. The titre of the released virus was determined at 12 hpi, (n = 3). **B)** Huh7 cells were infected with CHIKV (MOI 10) and expression levels of CHIKV nsP1 and capsid were determined by western blot at 24 hpi. **C)** CHIKV-infected Huh7 cells (MOI 2) were treated with decreasing concentrations of the Cl^-^ channel inhibitors; Ribavirin was used as control. The titer was determined at 12 hpi, (n ≥ 3). **D)** CHIKV was incubated with the compounds at 37°C (except for DEPC ≙ RT) for 1 hr prior to adsorption to Huh7 cells (MOI 2). The released CHIKV harvested and titred at 12 hpi, (n = 3). Error bars represent standard deviation. One-way ANOVA was performed to compare samples to untreated cells. * p≤0.05, ** p≤0.01, *** p≤0.001.

We first assessed well-characterized Cl^-^ channel blockers—including diisothiocyanostilbene-2,20-disulfonic acid (DIDS), 9-anthracene carboxylic acid (9-ACA), indyanyloxyacetic acid 94 (IAA-94) and 5-nitro-2-3-phenylpropylamino benzoic acid (NPPB) for their effects on CHIKV infection. For these assays, maximal non-toxic doses (MNTD) for human hepatoma cells (Huh7) were determined for each compound by MTT assay (S1 Fig). Next, Huh7 cells were infected with CHIKV (MOI 2) in the presence of the MNTD of each compound; at 12 hpi titers of released virus were determined. Treatment with NPPB resulted in an 18-fold reduction in CHIKV progeny compared to untreated cells, while treatment with DIDS and 9-ACA led to an 8-fold decrease in CHIKV titer (p ≤ 0.05) (Fig 1A). IAA-94 had no detectable effects. Ribavirin, shown previously to be active against CHIKV [27, 28], was included as a positive control and significantly inhibited production of CHIKV virions (56-fold decrease).

To confirm these findings, the expression of CHIKV nsP1 and capsid protein in the presence each Cl^-^ channel inhibitor were determined by western blot analysis. Fig 1B shows reduced expression of nsP1 and capsid in DIDS, 9-ACA and NPPB-treated cells compared to untreated/DMSO-treated cells and only in the presence of IAA-94 were nsP1/capsid levels unaffected. DIDS, 9-ACA, NPPB and Ribavirin were found to inhibit CHIKV replication in a dose dependent manner (Fig 1Ci–1Civ). This data further implies that functional Cl^-^ channels are required during the CHIKV lifecycle. The inhibitory effect on viral protein expression was consistent in cells infected with greater and smaller MOIs (S2 Fig). To exclude any direct virucidal activity of the compounds, CHIKV virions were incubated with the MNTD of each compound *in vitro* at 37°C for one hour prior to their dilution in media and infection onto untreated cells (Fig 1D). DEPC was included in this assay as a positive control, as it has previously been shown to modify histidine’s on CHIKV glycoproteins, preventing viral fusion [29]. Treatment with DIDS, 9-ACA, NPPB and Ribavirin had no significant effects on the CHIKV titer following direct virion treatment, implying that the compounds do not inactivate the viral particle. Taken together, these data provide the first reported requirement for DIDS-, 9-ACA- and NPPB-sensitive cellular Cl^-^ channels during the CHIKV lifecycle.

### Cl^-^ channels are required during post-attachment/internalization stages of the CHIKV lifecycle

**Fig 2:**
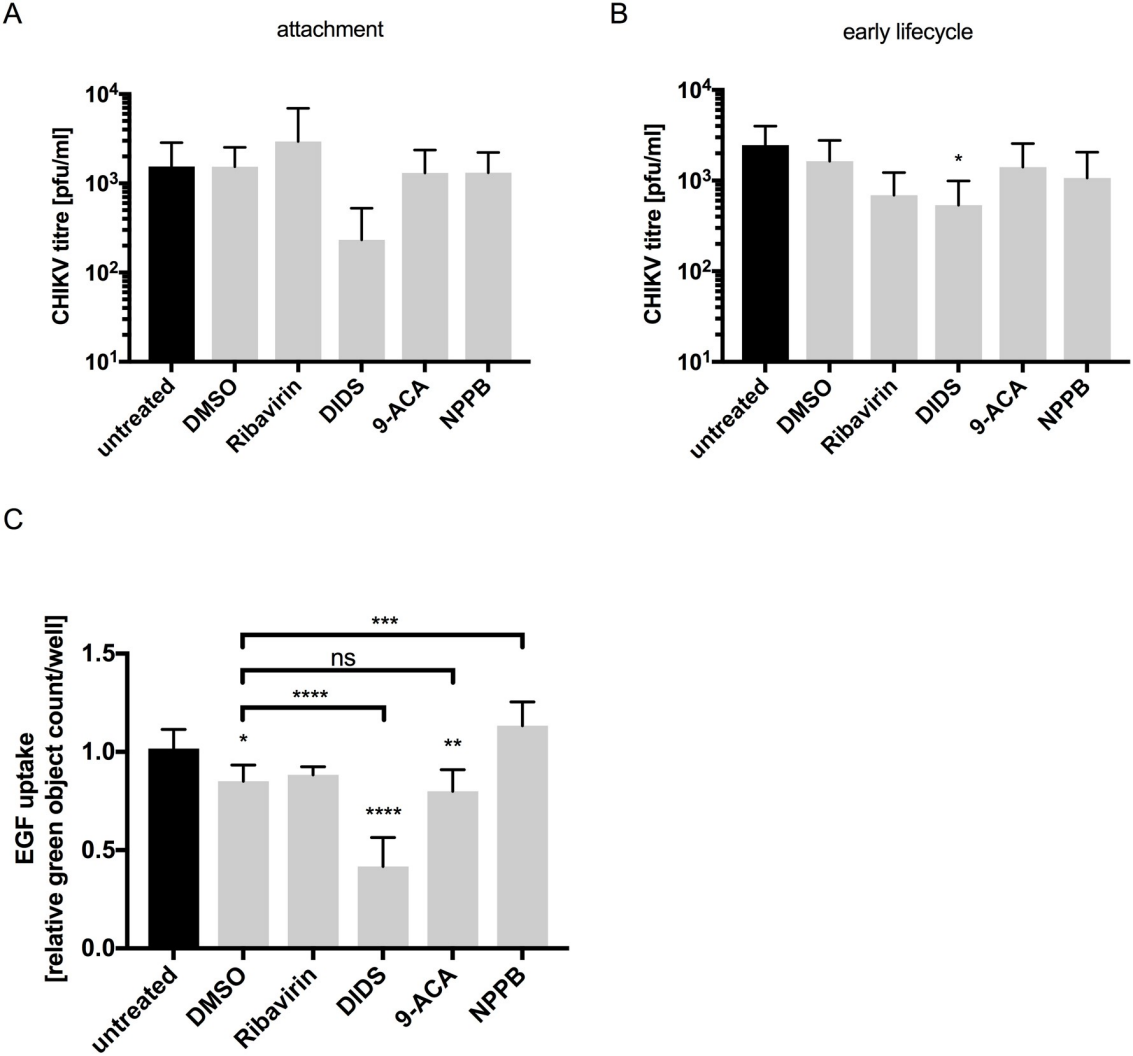
Cl^-^ channels are mainly required during post-attachment/early stages of the CHIKV lifecycle. **A)** Huh7 cells were treated with the Cl^-^ channel inhibitors for 1 hr prior to adsorption of CHIKV (MOI 2) at 4°C to facilitate attachment but prevent internalization of the virus into the cell. After removal of the virus, cells were returned to 37°C and titer of released CHIKV was determined at 24 hpi (n = 3). **B)** Huh7 cells were treated with compounds for 1 hr prior to adsorption of the virus. Virus was then removed and cells incubated with the compounds for one further hour. The titer of released CHIKV was determined at 12 hpi (n = 3). **C)** Huh7 cells were treated with the compounds for 1 hr at 37°C prior to addition of the Alexa Fluor 488 EGF complex. Cells were fixed after 45 mins and the green object count/well determined using the Incucyte Zoom (n = 3). The modest, but significant decrease in Alexa Fluor 488 EGF uptake upon treatment with the carrier control (DMSO) was controlled for in the analysis by comparing the samples to DMSO-treated samples in the one-way ANOVA. Error bars represents standard deviation. One-way ANOVA was performed to compare samples to untreated/DMSO cells. ns = not significant, *p≤0.05; ** p≤0. 01, *** p≤0.001, **** p≤0.0001.

We next sought to identify the stage of the CHIKV lifecycle that is sensitive to Cl^-^ channel inhibition. The CHIKV lifecycle begins with virus attachment to host cells mediated by the CHIKV E2 protein. To analyze cell attachment, CHIKV (MOI 2) was adsorbed onto cells at 4°C (to prevent virus internalization) for 1 hr in the presence of either DIDS, 9-ACA, NPPB or Ribavirin. Both virus and compound were then removed by rigorous washing and cells were warmed to 37°C to permit the internalization of attached virus particles. Virus infection efficiency was lower in this assay compared to infections carried out at physiological temperature. Thus, cell supernatants were assayed for infectious CHIKV progeny at 24 hpi (Fig 2A), rather than 12 hpi as in other assays. Treatment with 9-ACA, NPPB and Ribavirin had no influence on CHIKV titers, indicating that these compounds do not impact CHIKV E2 mediated cell surface attachment. DIDS had a modest but non-significant effect on virus binding, implying it may partially impact this lifecycle stage.

The first CHIKV particles fuse with endosomal membranes as early as 2 mins post-infection, making CHIKV entry a rapid process (12). Indeed it has been reported that almost all CHIKV particles escape the endosomal system within the initial 22 mins of infection. Following endosomal fusion, the viral capsid is released, genome is translated and replication complexes begin to form. We investigated the requirement of Cl^-^ channels during this early lifecycle stage by adsorbing CHIKV onto pre-treated cells for 1 hr at 37°C (MOI 2). Virus and compound were then removed and non-cell associated viruses were washed from cells. Cells were treated with each compound for a further 1 hr and after their removal, CHIKV titers were assessed at 12 hpi. As shown in Fig 2B, 9-ACA, NPPB and Ribavirin had no effect on CHIKV infection when treatment was limited to the early lifecycle stages. DIDS-treated cells, however, displayed reduced CHIKV titers (p ≤ 0.05), consistent with its modest effects on virus attachment. Some of the inhibitory effect associated with DIDS-treatment appeared to be non-specific and associated with general inhibition of clathrin-mediated endocytosis, since cells accumulated lower levels of Alexa Fluo 488 EGF following DIDS-treatment (Fig 2C). NPPB-treatment in this assay led to a small increase in EGF uptake by an as yet undescribed mechanism.

Taken together, these data indicate that neither 9-ACA or NPPB inhibit CHIKV at the stages of virus attachment or internalization, whilst DIDS inhibits CHIKV early attachment and internalization.

### Efficient CHIKV RNA replication requires functional cellular Cl^-^ channels

**Fig 3:**
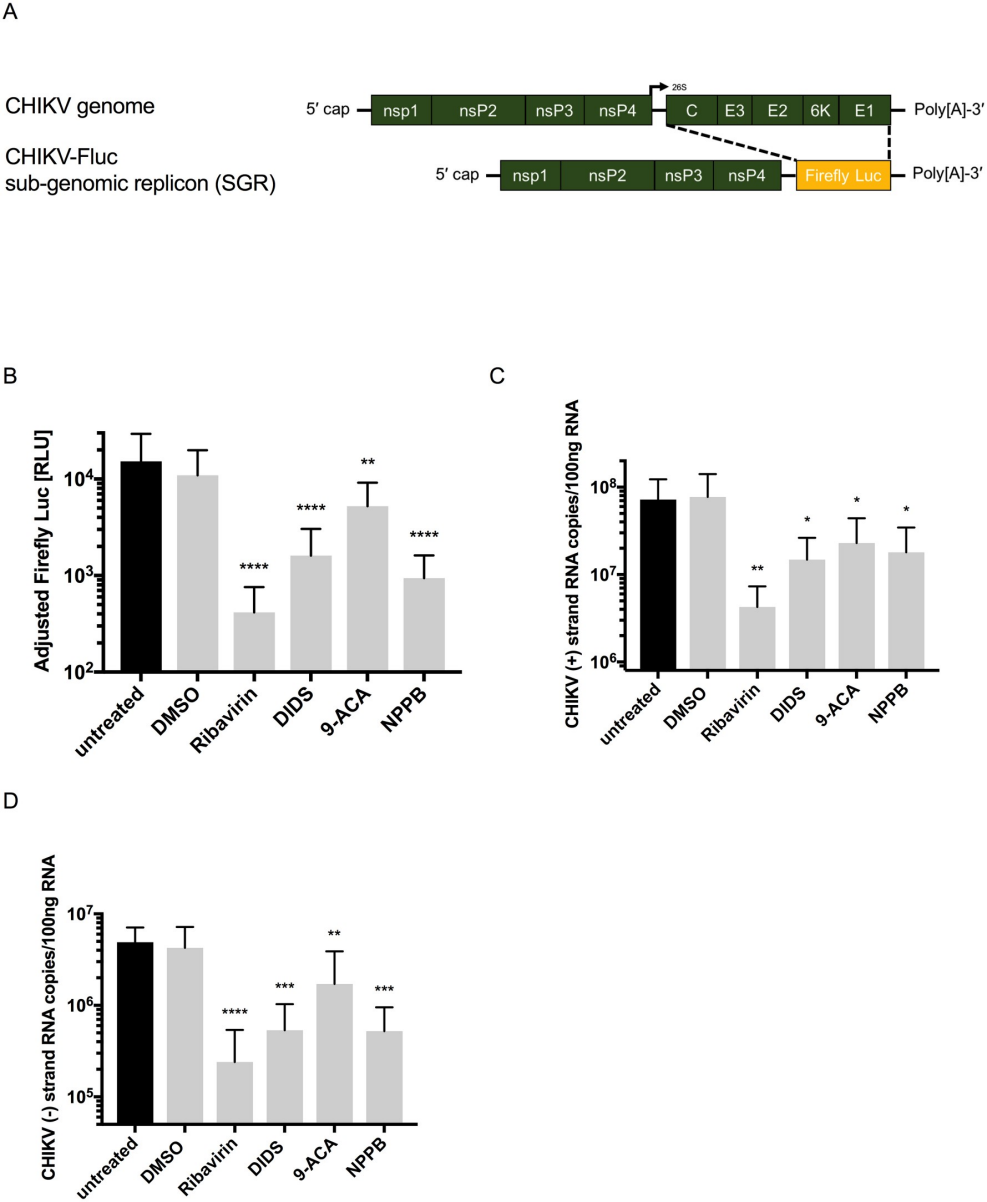
CHIKV genome replication requires functional Cl^-^ channels. **A)** Schematic of the CHIKV genome (top) and the CHIKV-Fluc SGR (bottom). **B)** Huh7 cells were transfected with the CHIKV-Fluc SGR and Rluc mRNA in the presence of Cl^-^ channel inhibitors. At 6 hrs post-transfection, Fluc and Rluc activities were determined and Fluc activities normalised to Rluc. **C and D)** Total RNA was extracted from Huh7 cells 6 hpi with CHIKV (MOI 2) and treatment with the Cl^-^ channel inhibitors. CHIKV positive-strand genomic **(C)** and minus-strand intermediate **(D)** RNA was quantified by reverse transcription followed by qPCR (n = 4). One-way ANOVA was performed to compare samples to untreated cells. Error bars represent standard deviation. ns = not significant, * p≤0.05, ** p≤0.01, *** p≤0.001, **** p≤0.0001.

We then investigated the effects of the Cl^-^ channel inhibitors on efficient CHIKV RNA replication using a CHIKV-Fluc SGR (Fig 3A) and Fluc luminescence as a proxy of CHIKV RNA synthesis. For these assays, cells were briefly pre-treated with the MNTD of DIDS, 9-ACA, NPPB or Ribavirin before co-transfection with the CHIKV-Fluc SGR and a 5’capped Rluc mRNA *in trans*, to control for differences in transfection efficiency. Cells were assessed for CHIKV replication by the measurement of luciferase activity 6 hrs post-transfection. NPPB-treatment significantly inhibited CHIKV-Fluc SGR replication 16-fold (p ≤ 0.0001) while DIDS-treatment led to a 9-fold decrease in CHIKV-Fluc SGR replication. As expected, ribavirin also significantly lowered CHIKV-Fluc SGR replication 37-fold (p ≤ 0.0001). 9-ACA treatment produced only a comparatively modest ~3-fold reduction (p ≤ 0.01) in CHIKV-Fluc SGR replication (Fig 3B). To confirm these findings, we quantified CHIKV genomic (Fig 3C) and intermediate complimentary minus-strand (Fig 3D) RNA copy number (Fig 3C) by ssqPCR. For these assays, cells were infected (MOI 2) in the presence of each Cl^-^ channel inhibitory compound and total RNA extracted at 6 hpi. Significantly lower levels of the CHIKV genomic RNA were observed in cells treated with DIDS (p ≤ 0.05), 9-ACA (p ≤ 0.05), NPPB (p ≤ 0.05) or Ribavirin (p ≤ 0.01) (Fig 3C), consistent with the SGR data (Fig 3B). Similarly, copy numbers of the intermediate complimentary minus-strand RNA were significantly reduced with DIDS- (p ≤ 0.001), 9-ACA- (p ≤ 0.01), NPPB- (p ≤ 0.001) and Ribavirin- (p ≤ 0.0001) treatment. From this data, we inferred that the efficiency of CHIKV genome replication is dependent on the function of Cl^−^ channels that are sensitive to DIDS, 9-ACA and NPPB–via either a direct role in replication of the CHIKV genome or indirectly through upregulation of ORF-1 translation.

### Cl^-^ intracellular channels (CLICs) facilitate CHIKV replication

**Fig 4:**
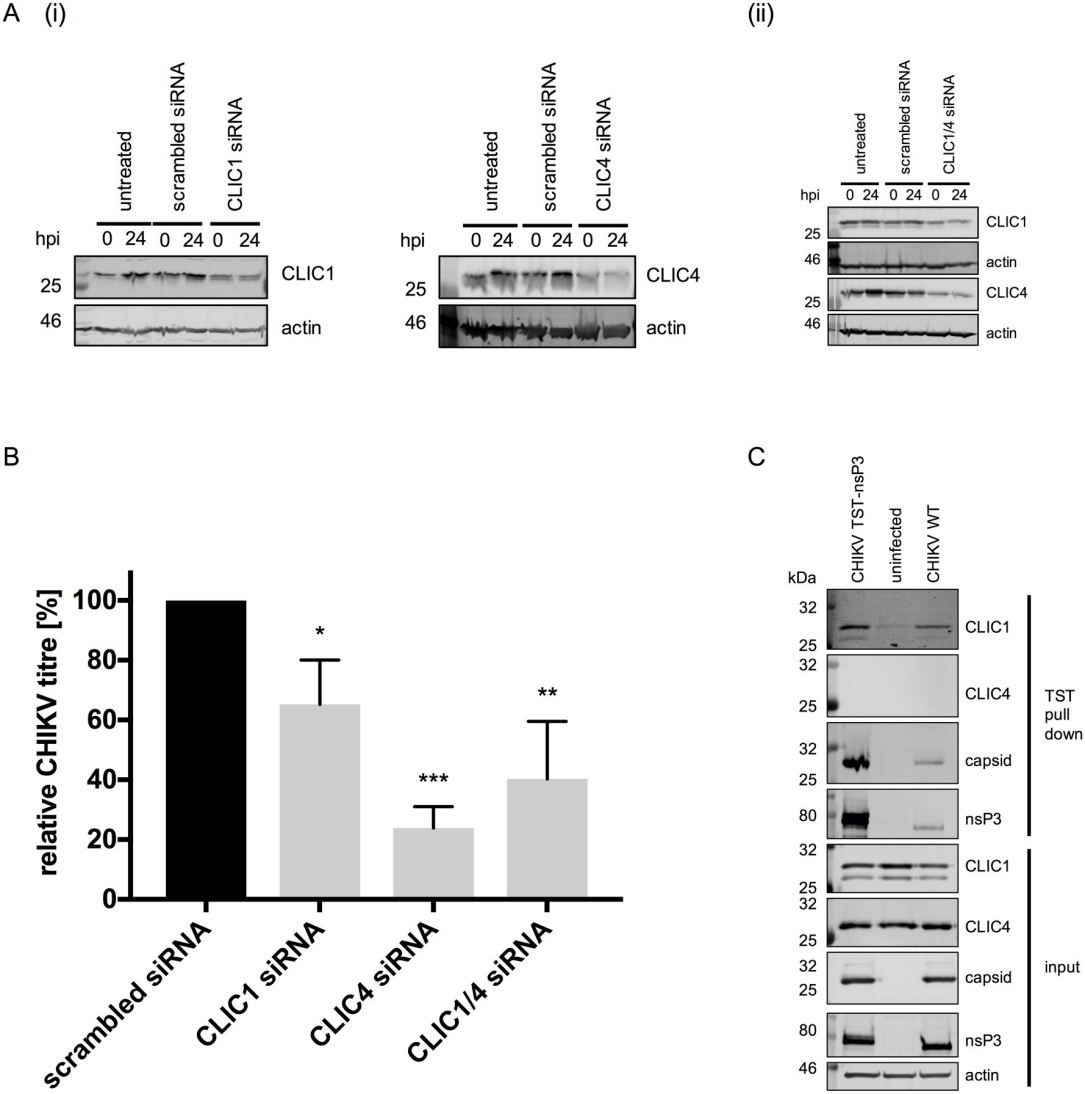
Chloride intracellular channels (CLICs) facilitate CHIKV replication. **A) (i)** CLIC1 and CLIC4, respectively, as well as **(ii)** CLIC1 and CLIC4 in combination, were knocked down by transfecting siRNA into Huh7 cells. **B)** At 48 hrs after initial siRNA transfection, cells were infected with CHIKV (MOI 10) and released CHIKV and titered at 24 hpi (n = 3). Viral titer was normalised to the scrambled siRNA control sample. One-way ANOVA was performed to compare samples to scrambled siRNA transfected cells. Error bars represent standard deviation. *p≤0.05; ** p≤0.01, *** p≤0.001. **C)** TST-tagged nsP3 from CHIKV infected (MOI 10) Huh7 cells (24 hpi) was pulled-down using Strep-Tactin Sepharose. Western blots were probed for nsP3 binding proteins.

All of the Cl^-^ channel compounds used in this study inhibit a broad range of cellular Cl^-^ channels. To date, ≥40 Cl^-^ channels have been identified that fall into six classes; 1) Cl^-^ intracellular channels (CLICs), 2) voltage-gated Cl^-^ channels (CLCs), 3) cystic fibrosis transmembrane conductance regulator (CFTR), 4) calcium-activated Cl^-^ channels (CaCCs), 5) ligand-gated Cl^-^ channels and 6) volume-regulated Cl^-^ channels (VRAC) [30, 31]. We focused on CLIC1 and CLIC4 as candidate channels, due to their known sensitivity to DIDS, 9-ACA and NPPB and involvement in other virus infections such as hepatitis C virus and Merkel cell polyomavirus [21, 32]. Initially, we confirmed by western blot that CHIKV infection did not induce expression of CLIC1 or CLIC4 (S3 Fig). CLIC1 and CLIC4 were then silenced using siRNA (Fig 4Ai), cells were then infected with CHIKV (MOI 10) and released virus titers determined at 24 hpi. Compared to control siRNA transfected cells, in those with reduced CLIC1 or CLIC4 expression levels, replication of CHIKV was significantly inhibited (p ≤ 0.05 and p ≤ 0.001, respectively) (Fig 4B), confirming a role for both CLIC1 and CLIC4 in efficient CHIKV replication. Co-silencing of both CLIC1 and CLIC4 (Fig 4Aii) did not result in synergistic inhibition of CHIKV replication (Fig 4B), implying that both channels are required for the same or a related stage in the virus lifecycle.

Given the inhibitory effects of chloride channel inhibition on CHIKV genome replication (Fig 3), we reasoned that the channels may form part of the virus replicase complex. To assess this, cells were infected with CHIKV twin-strep-tag nsP3 (CHIKV TST-nsP3) (MOI 10), which encodes a 28 amino acid TST-tag within the hypervariable domain of nsP3. At 24 hpi, cells were lysed and TST/strep-tactin affinity pull-downs performed—whereby the TST-tag of nsP3 selectively binds to Strep-Tactin Sepharose. Following elution, proteins interacting with CHIKV nsP3 were identified by western blot analysis. Negative controls for this analyses included cells infected with wild-type CHIKV (i.e. expressing untagged nsP3) and uninfected cells. We observed that untagged nsP3, capsid, and to lesser extent CLIC1, bound non-specifically to the Sepharose resin, albeit to low levels (Fig 4C). However, compared to both controls, enriched levels of CLIC1 were evident in complex with TST-nsP3—implying low levels of direct/indirect interaction between CLIC1 and nsP3. CLIC4 was not identified as an nsP3 interacting partner, suggesting an independent function outwith the CHIKV replicase complex. Previously published immunoprecipitation studies demonstrated an interaction between alphavirus capsid protein and nsP3 [33], which was included as a positive control to validate the pull-downs (Fig 4C). Taken together, these data suggest that CLIC1 interacts either directly or indirectly with nsP3, an essential component of the CHIKV replication machinery.

### Inhibition of Cl^-^ channels reduces CHIKV replication in invertebrate cells

**Fig 5:**
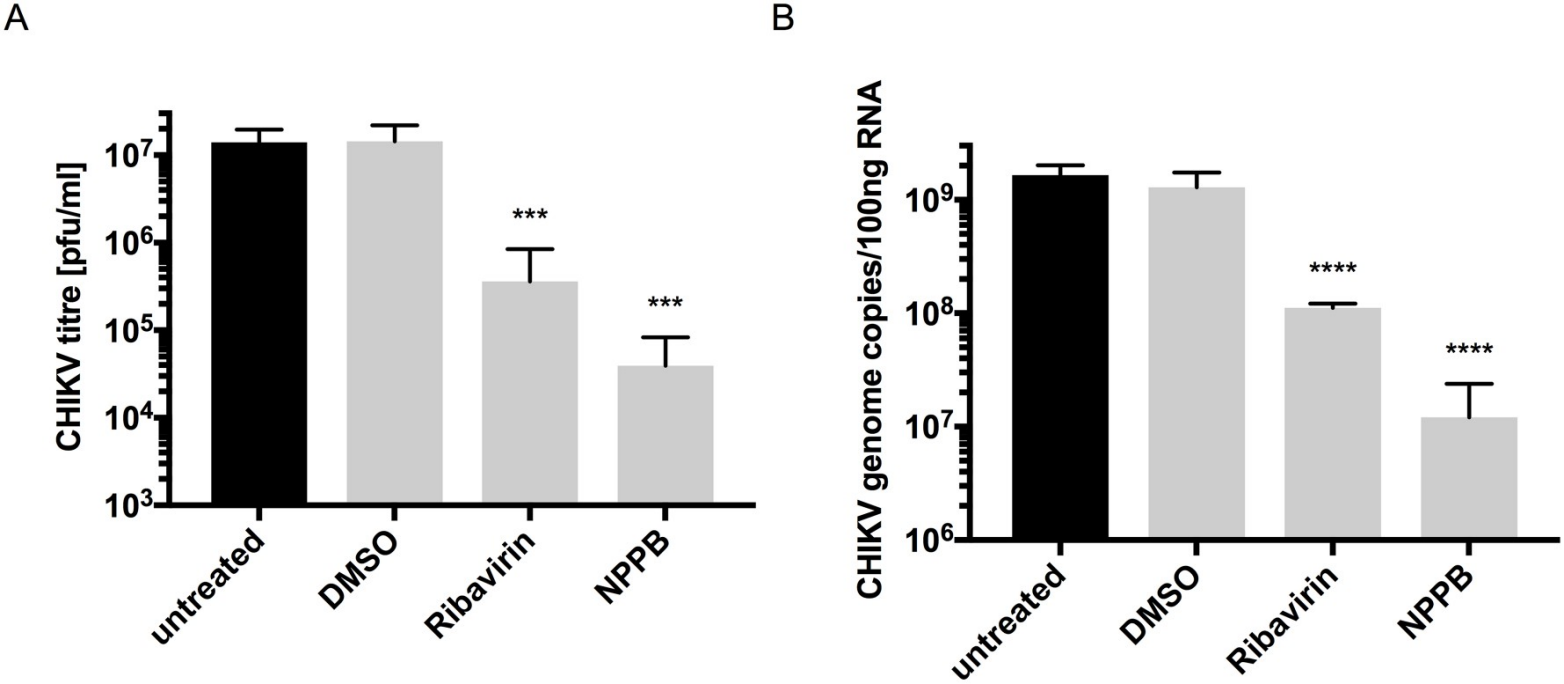
Inhibition of Cl^-^ channels reduces CHIKV replication in invertebrate cells. C6/36 cells were infected with CHIKV (MOI 2) in the presence of the MNTD of NPPB and Ribavirin, respectively. **A)** The viral titer (n = 3) and **B)** CHIKV RNA copy number (n = 3) were determined at 24 hpi. Error bars represent standard deviation. One-way ANOVA was performed to compare samples to untreated cells. *** p≤0.001, **** p≤0.0001.

As an arbovirus, CHIKV infects both humans and mosquitos. We thus investigated if the dependency of CHIKV on cellular Cl^-^ channels is limited to human cells or extends to those of its mosquito host. To assess this, C6/36 cells derived from the larva of *Ae*. *albopictus* were infected with CHIKV in the presence of the MNTD (S1B Fig) of NPPB, the Cl^-^ channel inhibitor that exhibited the most significant inhibitory effects on CHIKV replication in mammalian cells (Figs 1 and 3). A 357-fold reduction in CHIKV titer, following infection (MOI 2) of NPPB-treated C6/36 cells, was observed at 24 hpi (p ≤ 0.001), implying that Cl^-^ channels are required during the CHIKV lifecycle in mosquito host cells. Ribavirin-treatment, used as a positive control, led to a 39-fold decrease in CHIKV titer (p ≤ 0.001) (Fig 5A). The effects of the other Cl^-^ channel inhibitors (DIDS, 9-ACA and IAA-94) tested in mammalian cells, were also investigated in infections of C6/36 cells (MOI 2) (S4 Fig). Only DIDS-treatment led to a significant reduction in CHIKV replication at 24 hpi. As this could have been due to a similar non-specific effect as observed in Fig 2.—DIDS, 9-ACA and IAA-94 were not pursued further in C6/36 cell infection studies. By analogy to the data from mammalian cells, the effects of NPPB on viral genome replication were investigated using the CHIKV-Fluc SGR. Though the cytotoxic effect of CHIKV-Fluc SGR transfection in combination with NPPB-treatment prevented replicon analysis, CHIKV RNA copy numbers could still be determined by reverse transcription qPCR 24 hpi. Viral RNA copy numbers were reduced 137-fold in NPPB-treated cells (Fig 5B) and 15-fold in Ribavirin-treated cells (p ≤ 0.0001). Taken together, these data demonstrate that NPPB-sensitive Cl^-^ channels are involved in the CHIKV lifecycle in both human and mosquito host cells.

## Discussion

In this study we demonstrate that cellular Cl^-^ channels have a significant pro-viral role during CHIKV infection. Our data shows that Cl^-^ channel modulators inhibit efficient CHIKV RNA synthesis and that the intracellular Cl^-^ channels CLIC1 and CLIC4 are specifically required for CHIKV replication. CLIC1 was found in complex with CHIKV nsP3, albeit to low levels, suggesting a direct involvement in CHIKV replication complex formation or nsP3-mediated viral functions. For the first time, these data highlight the requirement of cellular ion channels during the CHIKV lifecycle.

Cl^-^ channels at the cell membrane regulate an array of cellular processes including cell-volume control, fluid transport and cell excitability. Intracellularly, Cl^-^ transport across organelle membranes regulates endosome, lysosome and Golgi acidification. We observed inhibitory effects for three out of four assessed Cl^-^ channel inhibitors on CHIKV replication in mammalian cells (Fig 1A).

The tested compounds do not display virucidal activity (Fig 1D) nor influence virus binding or the early stages of the viral lifecycle (Fig 2). Intriguingly, 9-ACA- and NPPB-sensitive Cl^-^ channels were required specifically for efficient RNA replication—as evidenced by the effect of the inhibitors on the CHIKV-Fluc SGR and copy number of the CHIKV genomic and intermediate minus-strand RNA (Fig 3). NPPB-sensitive Cl^-^ channels appear to have a conserved function in mosquito cells, as application of the inhibitor to infected C6/36 cells led to a reduction in CHIKV replication (Fig 5).

To identify specific Cl^-^ channels required for CHIKV replication, we focused on two CLIC family members, namely CLIC1 and CLIC4. CLIC proteins are small proteins (236–253 aa, with the exception of CLIC5B and CLIC6), harboring an N-terminal GST-like domain and a C-terminal alpha-helical domain (reviewed in [34]). CLICs are metamorphic proteins that reversibly alternate between soluble cytoplasmic and membrane-associated forms, by rearrangement of the GST-like fold under oxidative conditions. Consequently, CLICs are multifunctional, exerting GST-like enzymatic functions as well as functions in membrane trafficking, endosomal sorting and functioning as Cl^-^ channels. In Merkel cell polyomavirus infected cells, both CLIC1 and CLIC4, mediate small T antigen induced cell motility via their Cl^-^ channel activity [32]. In this study, CLIC1 and CLIC4 silencing inhibited CHIKV replication and, given that these effects mirrored those of the Cl^-^ modulators, we reasoned that this effect was likely due to Cl^-^ channel activity. Interestingly, we identified CLIC1 as a potential interacting partner of nsP3—an essential component of the CHIKV replication complex. We thus speculate that CLIC1 is in its membrane-inserted conformation and interacts with nsP3 and/or other ns-protein(s) bound to nsP3 as part of the replicase, to ensure optimal replication conditions within this membrane imbedded complex. These functions may align with the known roles of intracellular Cl^-^ function, namely the regulation of organelle pH to maintain complex stability and genome integrity. In addition, isolation of CHIKV replication complexes has shown that all proteins needed for viral genome replication are present in the membrane fraction, and no soluble proteins are required [35], further supporting a role for membrane associated CLIC1. The double knock down of CLIC1 and CLIC4 did not have a synergistic effect on inhibiting CHIKV replication (Fig 4B) and CLIC4 was not observed to directly interact with nsP3. These results imply that, although required for efficient CHIKV replication, CLIC4 functions through an alternative mechanism or different stage in a pathway to CLIC1.

Using a combination of broad acting inhibitors and siRNA screens, the involvement of several Cl^-^ channel/transporter proteins in the hepatitis C virus lifecycle has previously been reported [21], with CLC2, CLC3, CLC5, CLC7 being specifically required for genome replication. A Cl^-^ channel from the same family, CLC6, has been identified as a pro-viral factor in a genome-wide loss of function screen performed with a CHIKV reporter virus [22]. This raises possibility that further Cl^-^ channels are involved in the replication of the CHIKV genome or other stages of the virus lifecycle. For example, it is conceivable that Cl^-^ channels not targeted by the compounds used in this study, or other ion channels, may play a role in post-entry events, leading up to genome replication—i.e. trafficking or uncoating as observed for e.g. Bunyamwera virus [16, 17].

Anion channels have been shown to be important in other viral systems. Cl^-^ channels were identified as part of the Semliki Forest virus, that is closely related to CHIKV, replication complex by quantitative proteomics [36], supporting our CLIC1/nsP3 findings. In addition, the replication of Tomato bushy stunt virus (family *Tombusviridae*) was shown to depend on Cl^—^proton exchanger function [37]. Gef1p Cl^-^silencing, a homologue of the mammalian CLC proteins in yeast, inhibited replication through its downstream effects on Cu^2+^ homoeostasis, inhibiting the functionality of the viral replicase. Direct interaction of the voltage-dependent anion channel 1 with VP1 and VP3 of infectious bursal disease virus (family *Birnavirdae*) was shown to stabilize the ribonucleoprotein complex, allowing full activity of the viral polymerase [38]. It may be possible that CLIC1, as an nsP3 interacting protein, is required for CHIKV genome replication for a similar mechanism.

Interestingly, CLIC-dependent Cl^-^ efflux has been shown to act during NLRP3 inflammasome activation and signaling [39] and it has recently been shown that the NLRP3 inflammasome is activated in CHIKV infected humans [40]. A small molecule inhibitor of the inflammasome abrogated inflammatory pathology in mice without influencing the CHIKV titer. This could imply that CLIC Cl^-^ channel function is also involved in the inflammatory response to CHIKV infection, in addition to its role in efficient CHIKV genome replication. Inhibitors specific to CLIC1 and CLIC4 may hold potential as both CHIKV antivirals and inhibitors of the CHIKV inflammatory response. Specific inhibitors would exclude possible off-target effects caused by the currently available broad acting Cl^-^ channel inhibitors.

In conclusion, for the first time this study identifies Cl^-^ channels as essential host cell factors for efficient CHIKV replication and in conjunction with other recent studies highlights the significance of ion channel modulation as a druggable target to inhibit virus infection. Notably, we show that the requirement for cellular Cl^-^ channels is conserved between human and mosquito host cells and it is likely that other Cl^-^ channels are required at various stages of the CHIKV lifecycle. In general, Cl^-^ channels may represent a potential target for the development of antivirals acting against a broad variety of (arbo-)viruses and encouragingly Cl^-^ channel inhibitors are in clinical or pre-clinical use [30], potentially facilitatatig the development of Cl^-^ channel specific compounds for future anti-CHIKV strategies.

## Supporting information

S1 Fig Maximal non-toxic doses (MNTD) of Cl^-^ channel inhibitors and Ribavirin. Huh7 **(A)** and C6/36 cells **(B)** were incubated with increasing concentrations of Cl^-^ channel inhibitors, Ribavirin and carrier (DMSO) only. Cell viability was determined by MTT assay after 6 hrs and 24 hrs of treatment, respectively (n ≥ 3). Arrows indicate the maximal non-toxic doses. Error bars represent standard deviation. One-way ANOVA was performed to compare samples to untreated cells.
(TIF)

S2 Fig Inhibition of Cl^-^ channels decreases CHIKV protein expression after infection at different MOIs. Huh7 cells were infected with CHIKV at an MOI 10 (**A**) and MOI 0.1 (**B**), respectively, and the intracellular expression levels of CHIKV nsP1 and capsid determined by western blot at 24 hpi.
(TIF)

S3 Fig CHIKV infection does not affect expression of pro-viral proteins CLIC1 and CLIC4. Huh7 cells were infected with CHIKV (MOI 2) and cellular proteins analysed by western blot at 24 hpi. CLIC1 (**A**) and CLIC4 (**B**) protein levels were not altered by infection with CHIKV.
(TIF)

S4 Fig Effect of Cl^-^ channel inhibitors on CHIKV replication in invertebrate cells. C6/36 cells were infected with CHIKV (MOI 2) in the presence of the MNTD of DIDS, 9-ACA and IAA-94 and Ribavirin. The viral titer was determined at 24 hpi (n = 3). Error bars represent standard deviation. One-way ANOVA was performed to compare samples to untreated cells. *** p≤0.001, **** p≤0.0001.
(TIF)

S1 Table Primer sequences for the reverse transcription and quantitative PCRs for CHIKV strand-specific detection. (DOCX)
