# Supplementary figures and images for: Chikungunya virus requires cellular chloride channels for efficient genome replication
Source: PLoS Negl Trop Dis. 2019 Sep 4;13(9):e0007703. doi: 10.1371/journal.pntd.0007703 (PMC6746389; doi:10.1371/journal.pntd.0007703)

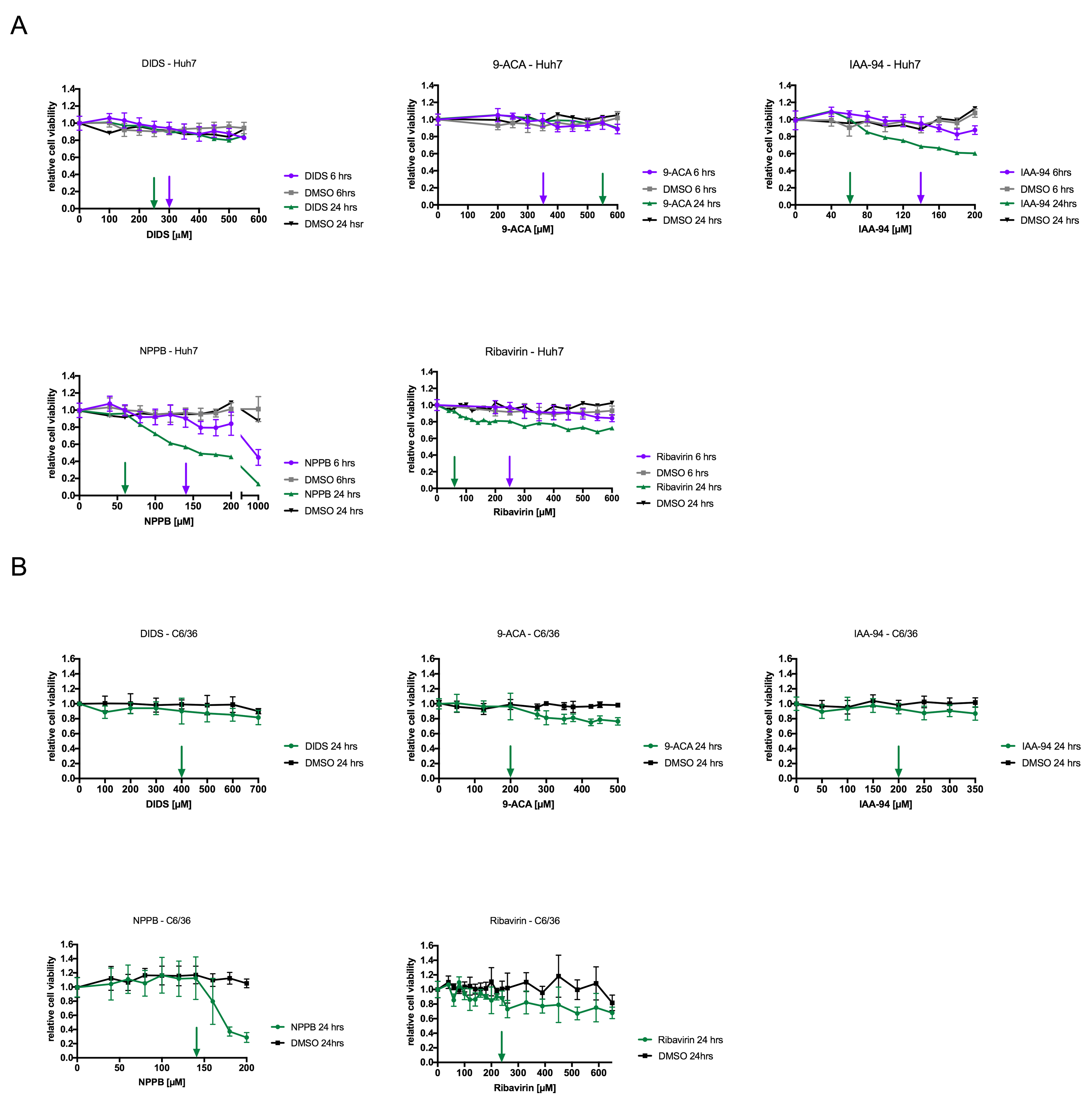

Supplement: S1 Fig — Huh7 (A) and C6/36 cells (B) were incubated with increasing concentrations of Cl- channel inhibitors, Ribavirin and carrier (DMSO) only. Cell viability was determined by MTT assay after 6 hrs and 24 hrs of treatment, respectively (n ≥ 3). Arrows indicate the maximal non-toxic doses. Error bars represent standard deviation. One-way ANOVA was performed to compare samples to untreated cells. (TIF) [file pntd.0007703.s001.tif]

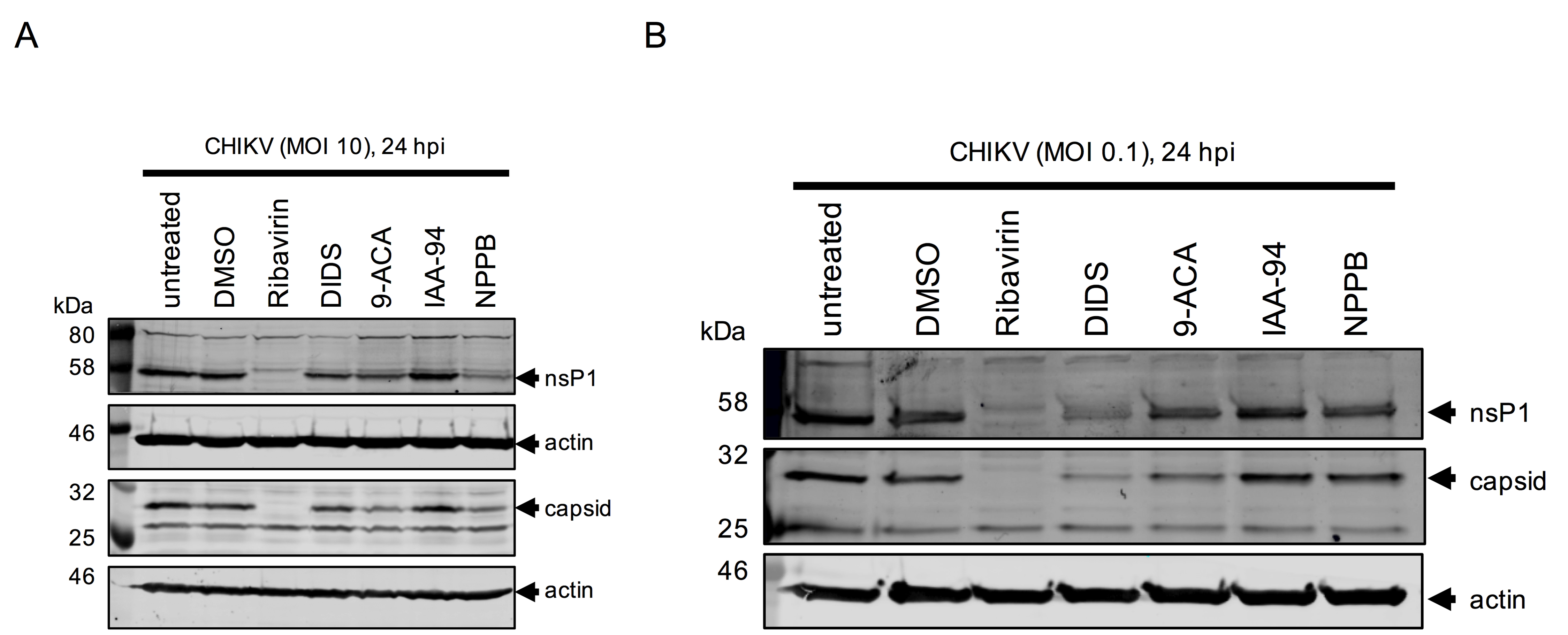

Supplement: S2 Fig — Huh7 cells were infected with CHIKV at an MOI 10 (A) and MOI 0.1 (B), respectively, and the intracellular expression levels of CHIKV nsP1 and capsid determined by western blot at 24 hpi. (TIF) [file pntd.0007703.s002.tif]

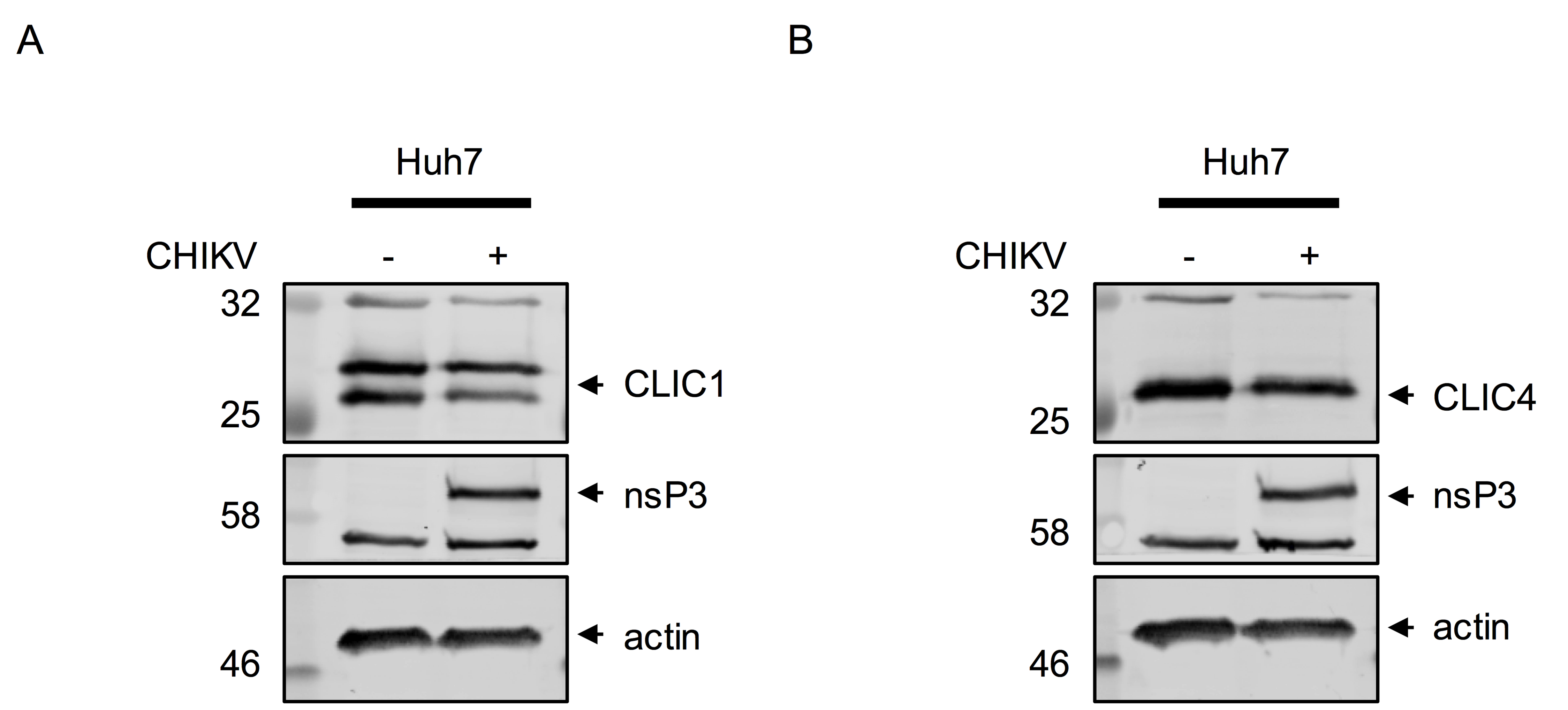

Supplement: S3 Fig — Huh7 cells were infected with CHIKV (MOI 2) and cellular proteins analysed by western blot at 24 hpi. CLIC1 (A) and CLIC4 (B) protein levels were not altered by infection with CHIKV. (TIF) [file pntd.0007703.s003.tif]

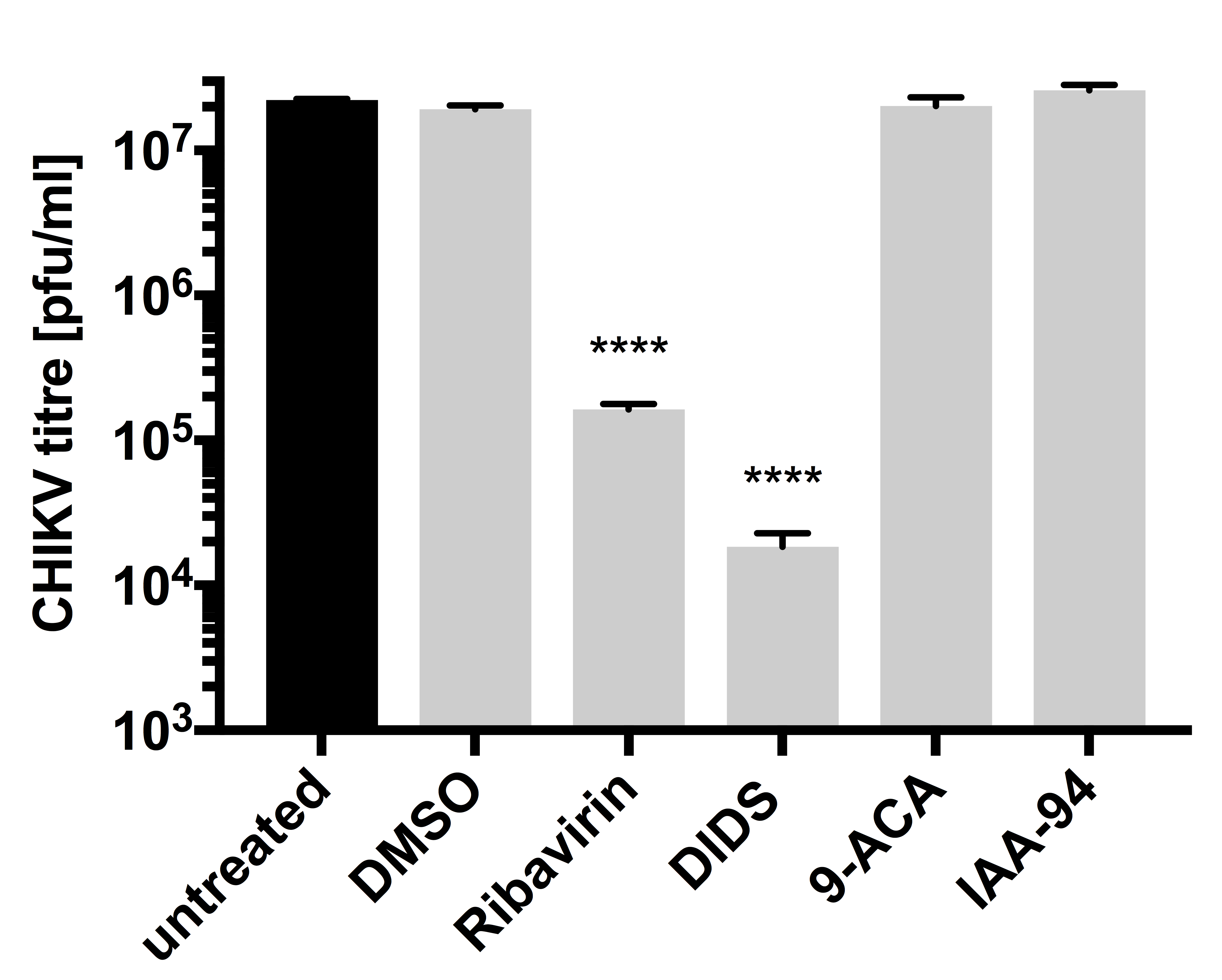

Supplement: S4 Fig — C6/36 cells were infected with CHIKV (MOI 2) in the presence of the MNTD of DIDS, 9-ACA and IAA-94 and Ribavirin. The viral titer was determined at 24 hpi (n = 3). Error bars represent standard deviation. One-way ANOVA was performed to compare samples to untreated cells. *** p≤0.001, **** p≤0.0001. (TIF) [file pntd.0007703.s004.tif]
