# Supplementary material for: Chikungunya virus requires cellular chloride channels for efficient genome replication
Source: PLoS Negl Trop Dis. 2019 Sep 4;13(9):e0007703. doi: 10.1371/journal.pntd.0007703 (PMC6746389; doi:10.1371/journal.pntd.0007703)
Supplement: S1 Table — (DOCX) [file pntd.0007703.s005.docx]

| CHIKV (-) strand detection | PCR | Primer sequence (5’-3’) |
| --- | --- | --- |
| CHIKV FT tag T | reverse transcription | GGC AGT ATC GTG AAT TCG ATG CGA CAC GGA GAC GCC AAC ATT |
| Tag T | quantitative | GGC AGT ATC GTG AAT TCG ATG C |
| CHIKV R T | quantitative | AAT AAA TCA TAA GTC TGC TCT CTG TCT ACA TGA |
| CHIKV (+) strand detection | **PCR** | **Primer sequence (5’-3’)** |
| CHIKV RT tag T | reverse transcription | GGC AGT ATC GTG AAT TCG ATG CGT CTG CTC TCT GTC TAC ATG A |
| CHIKV F T | quantitative | AAT AAA TCA TAA GAC ACG GAG ACG CCA ACA TT |
| Tag T | quantitative | see above |

**S1 Table: Primer sequences for the reverse transcription and quantitative PCRs for CHIKV strand-specific detection.**
